# Eutrophication and predator presence overrule the effects of temperature on mosquito survival and development

**DOI:** 10.1371/journal.pntd.0006354

**Published:** 2018-03-26

**Authors:** Maarten Schrama, Erin E. Gorsich, Ellard R. Hunting, S. Henrik Barmentlo, Brianna Beechler, Peter M. van Bodegom

**Affiliations:** 1 Institute of Environmental Sciences, Leiden University, Leiden, The Netherlands; 2 Naturalis Biodiversity Centre, Leiden, The Netherlands; 3 Department of Biology, Colorado State University, Fort Collins, Colorado, United States of America; 4 Department of Biomedical Sciences, College of Veterinary Medicine, Oregon State University, Corvallis, Oregon, United States of America; Egerton University, KENYA

## Abstract

Adequate predictions of mosquito-borne disease risk require an understanding of the relevant drivers governing mosquito populations. Since previous studies have focused mainly on the role of temperature, here we assessed the effects of other important ecological variables (predation, nutrient availability, presence of conspecifics) in conjunction with the role of temperature on mosquito life history parameters. We carried out two mesocosm experiments with the common brown house mosquito, *Culex pipiens*, a confirmed vector for West Nile Virus, Usutu and Sindbis, and a controphic species; the harlequin fly, *Chironomus riparius*. The first experiment quantified interactions between predation by *Notonecta glauca* L. (Hemiptera: Notonectidae) and temperature on adult emergence. The second experiment quantified interactions between nutrient additions and temperature on larval mortality and adult emergence. Results indicate that 1) irrespective of temperature, predator presence decreased mosquito larval survival and adult emergence by 20–50%, 2) nutrient additions led to a 3-4-fold increase in mosquito adult emergence and a 2-day decrease in development time across all temperature treatments, 3) neither predation, nutrient additions nor temperature had strong effects on the emergence and development rate of controphic *Ch*. *riparius*. Our study suggests that, in addition to of effects of temperature, ecological bottom-up (eutrophication) and top-down (predation) drivers can have strong effects on mosquito life history parameters. Current approaches to predicting mosquito-borne disease risk rely on large-scale proxies of mosquito population dynamics, such as temperature, vegetation characteristics and precipitation. Local scale management actions, however, will require understanding of the relevant top-down and bottom-up drivers of mosquito populations.

## Author summary

Human actions have strongly altered ecosystems worldwide, through climate change, eutrophication, and biodiversity loss. The consequences of these global changes for mosquito populations could have important implications for mosquito-borne infections. Previous studies have focused on the effects of temperature from climate change, but we lack a comprehensive understanding of how ecological factors related to global change influence mosquito populations. To this end, we carried out two mesocosm experiments with the common brown house mosquito, a vector for West Nile Virus, Usutu and Sindbis. The first experiment tested how the interaction between predation and temperature affected mosquito emergence from larvae to adults; the second experiment tested how the interaction between nutrient addition and temperature affected mortality and emergence. Our results show that predator presence decreased mosquito survival and emergence, whereas nutrient additions led to an increase in emergence and a decrease in development time. Temperature and competition had no major impact. Our study suggests that, in addition to effects of climate, ecological drivers can have strong effects on mosquito populations known to transmit disease.

## Introduction

Associations between anthropogenic pressures, disease risk and vector ecology are particularly strong for mosquito-borne infections [1–4]. To date, existing predictive maps of disease risk almost exclusively focus on large-scale drivers of mosquito populations, such as temperature, precipitation, and large scale vegetation properties [5–7]. These efforts have been fuelled by observed and predicted changes of the Earth’s climate [e.g., 8,9]. While temperature has indeed been shown to be a key determinant of mosquito development, survival, and fitness [9–14] it is often not fully appreciated that mosquitoes inhabit complex ecosystems and are exposed to a myriad of local biotic and abiotic factors that likely influence the dynamics of mosquito populations [15–18]. These factors operate on various scales, ranging from local-level pressures (e.g. pesticides, eutrophication) to regional (e.g. land use change) and global scales (e.g. climate change). Human activities are known to strongly alter these biotic and abiotic factors through nutrient additions, biodiversity declines and climate change [19]. Understanding how these biotic and abiotic factors in turn influence mosquito-borne disease risk requires quantifying how they interact to influence mosquito population dynamics.

Local mosquito population dynamics are mainly controlled by bottom-up (food availability) and top-down forces (predator abundance) [20–23]. Work by Hagstrum and Workman (1971) [22] suggests that temperature and food availability can jointly impact larval development rates (*Culex tarsalis)*. Temperature-dependent development rates were only observed in treatments with high food availability. Similarly, the effect of predators on mosquito populations may also be mediated by biotic and abiotic factors[17,21,24], such as eutrophication, the presence of controphics as alternative prey, habitat structure and pesticide concentrations. However, our current understanding of the factors driving mosquito populations are based on experiments that were carried out under highly simplified lab conditions devoid of abiotic variability and species interactions [8,9,25]. The relevance of this work under natural environmental conditions as well as the relative importance of the drivers for mosquito populations therefore remains unknown.

In this study, we used an outdoor mesocosm setup with *Culex pipiens*, a confirmed vector for West Nile virus, Usutu and Sindbis, to experimentally test the influence of three likely drivers of mosquito populations, representing three common anthropogenic pressures. Specifically, we manipulated nutrient concentrations, the presence or absence of predators and temperature to explore the consequences of eutrophication, biodiversity loss and climate change on mosquito population dynamics.

## Methods

### Experimental setup

Two mesocosm experiments were carried out in the experimental garden at the Hortus Botanicus of the University of Leiden, the Netherlands. The two experiments focused on role of temperature in conjunction with predation of larval mosquito populations or eutrophication of mosquito populated waters. Both experiments were conducted in 65-litre polyethylene tubs filled with 12 litres of rain water, which were set up in a semi latin-square design. In order to prevent excessive heating, each mesocosms was placed into the ground so its rim was approximately ten cm above the surface.

To allow for natural colonization of dipterans and standardized timing in the start of the experiment, the mesocosms were left open for 24 hours prior to both experiments. Within a single night, all mesocosms were colonized by two common Diptera species; *Culex pipiens*, a common mosquito species and *Chironomus riparius*, a controphic non-biting midge. To standardize the experimental settings, egg rafts were redistributed such that each mesocosm received two egg rafts of *Cx*. *pipiens* [in total equalling appr. 440 eggs; 26] and one egg raft of the harlequin fly *Ch*. *riparius* [equalling appr. 500 eggs; 27]. These densities were selected based on being within the observed range for *Cx*. *pipien*s, which varies widely under natural conditions [28]. Although there may be some variation in the number of eggs per raft, this is unlikely to influence the results because the egg rafts were randomly redistributed over the treatments. Preliminary experiments at this location showed that these two species typically colonize this type of habitat. To confirm that only these two species colonized our mesocosms, keys by Cranston et al. (1987) [29] for Culicidae and Langton (1984) [30] for Chironomidae were used. In the first experiment, four mesocosms were additionally colonized by herbivorous beetles, which we removed at the onset of the experiment. All mesocosms were covered with 50% shade cloth nets to prevent heating and animal escapes or introductions.

Both experiments used multiple temperature scenarios, for which aquarium heaters were used (50W, Aquadistri UK Ltd). The heaters were set at 24, 28 and 32°C in the first experiment and 18, 22, 26, 30°C in the second experiment. Allowing for fluctuating day-night temperature regimes, heaters were only switched on during the daylight hours between 6AM and 10PM, which represent the minimum and maximum daily temperature in the time of year that the experiments were carried out (S1 Fig). To monitor the temperature regimes, the temperature of each mesocosm was measured every 7 days using a portable hq 40d electronic multi-parameter meter (Hach Ltd, Colorado, US) at 6:00 AM (night temperature) and 12:00 PM (day temperature). The same device was used to record pH and electrical conductivity (EC), which were measured on a weekly basis. The average mesocosm temperature in this experiment was calculated as (16*measured day max temperature [measured at 12:00] + 8*minimum night temperature [measured at 6:00]) / 24 (Table 1), where 16 and 8 represent the daylight hours and night time hours respectively. This resulted in the following mean temperatures which are presented in the remainder of this manuscript: exp. 1; 22.7, 25.3 and 28.1°C; exp. 2: 22.1, 24.1, 26.1 and 26.8°C. Furthermore, no extra food was added to any of the mesocosms to mimic rainwater fed systems and ensure consistent and realistic nutrient concentrations.

**Table 1:** Counts and standard error (SE) of emerged adults, larvae and pupae of *Cx*. *pipiens* and *Ch*. *riparius* at the termination of experiment 1. The p-values under temperature effect and predation effect display the results of hypothesis tests for the effects of temperature category and predation on each response.

Scientific name	Parameter	Predation treatment	Temp1 22.7°C	SE	Temp2 25.3°C	SE	Temp3 28.1°C	SE	Temperature effect	Predator effect
*Cx*. *pipiens*	# Larvae surviving	N	85.1	± 40.8	69.6	± 21.2	31.3	± 13.8	ns	**p = 0.02**
* *		Y	42.0	± 10.6	18.9	± 8.9	21.3	± 10.6		
	# Pupae surviving	N	8.0	± 3.3	16.6	± 5.0	11.0	± 6.3	ns	**p = 0.02**
		Y	0.9	± 0.3	2.9	± 1.9	8.0	± 3.9		
	# Adults emerged	N	28.3	± 6.9	24.4	± 7.8	23.3	± 6.6	ns	**p = 0.02**
		Y	13.4	± 4.3	13.1	± 5.1	16.4	± 4.1		
*Ch*. *riparius*	# Larvae surviving	N	35.3	± 6.8	46.9	± 16.4	37.0	± 11.1	ns	**p = 0.005**
		Y	18.1	± 3.7	27.7	± 13.3	7.9	± 1.6		
	# Adults emerged	N	13.3	± 7.0	9.7	± 5.6	14.9	± 7.5	ns	ns
* *		Y	14.7	± 3.2	8.0	± 2.6	15.1	± 6.0		

#: number; ns: not significant; Temp: temperature in degrees Celcius. P -values were calculated based on a two-way ANOVA with square-root transformed response variables parameters for temperature, predation, and their interaction. No interaction terms were significant at α = 0.05.

### Experiment 1: Effects of predation on larval development rate, mortality and emergence

The first experiment was conducted between 15 May and 20 June 2016 in 42 mesocosms (S2 Fig). Three temperature scenarios with and without predators resulted in six treatments. Each treatment contained 7 replicate mesocosms, which were set up in a modified latin square design (S2 Fig). The effects of predation were investigated by adding one adult *Notonecta glauca* (Hemiptera: Notonectidae, collected on the same day from a natural population in a nearby pond within a natural population) to half of the mesocosms, five days after the experiment started. All *Notonecta glauca* individuals were added 8 days after the experiment started when all mesocosms had 2^nd^ instar larvae. The temperature regimes were set immediately following egg raft redistribution and predator addition. Two of the most important ecological factors affecting predation that should be considered when designing predation experiments are the predators’ dietary preference for mosquitoes and the abundance of alternative prey for the predators [17]. *Notonecta glauca* is a common aquatic predator in Europe and is known for its ability to colonize new habitats [31]. Furthermore, *N*. *glauca* is a visual hunter and confirmed predator of *Cx*. *pipiens* (S1 Table, S3 Fig) and *Ch*. *riparius* [31].

The effect of the treatments (predation and temperature) on three aspects of mosquito ecology were quantified: the cumulative number of emerged adult mosquitoes after 36 days, the eventual number of surviving mosquito larvae and the number of surviving mosquito pupae after 36 days. These dependent variables were uncorrelated and analysed separately. We distinguished between pupae and larvae because the experiment was terminated before all mosquitoes emerged, and we suspected predators to have stronger negative effects on pupae than on larvae because of their relative immobility. For Chironomids, only the number of emerged adults and survival of larvae were determined after 36 days. To quantify adult emergence of both *C*. *pipiens* and *C*. *riparius*, 10x10 cm Pherocon (Threce Adair, OK, US) sticky fly paper sheets with a general insect attractant were fitted below the top net of each mesocosm. These were replaced twice a week and all emerged adult mosquitoes (both species) were counted subsequently. This is a low invasive, unbiased method to determine emergence [32]. To quantify pupal and larval survival, the number of larval and pupal dipterans of both species remaining and alive after 36 days were counted. To count the remaining *Cx*. *pipiens* pupae and larvae, mesocosms were emptied by filtering the water using a 0.5 mm dipping net. For *Ch*. *riparius*, only the remaining larvae were counted.

### Experiment 2: Effects of eutrophication on larval development rate, mortality and emergence

The second experiment, focusing on the effect of eutrophication was conducted between 18^th^ of August and the 15^th^ of October 2016. It used a modified latin square design with 48 mesocosms and six replicates per treatment (S2 Fig). Eutrophication and temperature treatments (22.1, 24.1, 26.1 and 26.8°C), were initiated immediately following egg raft redistribution. Eutrophication treatments were applied to half of the mesocosms. An addition of 6.16 mL of soluble plant feed (Nitrogen:Phosphorus:Potassium 7:4:7; Pokon Naturado, The Netherlands) was added to half of the mesocosms to a final concentration of 9.0 mg inorganic nitrogen and 6.4 mg inorganic phosphorus per litre. These are typical values for stagnant, eutrophic, freshwater bodies [33]. The other half of the mesocosms received, as a control, a similar amount of untreated rain water. The numbers of larvae of both species were assessed on day 8 of the experiment, by gently filtering the entire volume of each mesocosm through a 0.5 mm sieve. This number was used for the larval development rate and survival calculations in this experiment. Emergence of the first *Cx*. *pipiens* was observed fourteen days after the experiment started, after which the emergence of adult mosquitoes and chironomids from all mesocosms was recorded daily (between 6 and 9 AM), using a manual aspirator, which was a much quicker method than the sticky trap for daily collections. Newly emerged mosquitoes were sexed and counted. Mean larval development rate was calculated as follows: 1/(average number of days between egg and emergence) and adult survival was calculated as follows: (number of emerged adults)/(number of larvae at day 8). To examine the effect of the nutrient addition on food availability, we measured the electrical conductivity (EC) and pH on a weekly basis. Electrical conductivity was used as a measure for the nutrient status [34]. because it reflects the abundance of microorganisms which compose the primary food for mosquito larvae [17]. Additionally, thirty days after the experiment started, chlorophyll A content was determined in each of the mesocosms. For this analysis, a subsample of 15 ml was collected from each of the mesocosms. These samples were filtered onto a Whatmann GF/F filter. Next, the filter was dissolved in 5 ml 90% acetone and allowed to break-down the algal cells for 20 hours at -20°C. Subsequently, samples were centrifuged at 1000G for 15 min at 4°C and supernatants were measured for absorbance at 620 nm using a plate reader. The experiment was terminated after 56 days when there were no more adults emerging from the mesocosms for 2 days. A single replicate mesocosm was colonized by *Daphnia magna* and excluded from further analysis.

### Data handling and statistics

First, the effect of the various temperatures in both experiments, top-down and bottom-up treatments on abiotic parameters were explored. Differences in mean day temperature and mean night temperature were tested with a one way ANOVA and a post-hoc Tukey test. The effect of temperature treatments and eutrophication treatments in experiment 2 on biotic (chlorophyll A) and abiotic (pH, EC) variables were tested using linear models, where temperature was a categorical variable and eutrophication was a binomial variable. For the number of emerged adults, number of surviving larvae and number of surviving pupae at day 35 of *Culex* and *Ch*. *riparius*, linear models with type III sum of squares were used to test the effects of temperature, predator presence and their interaction for experiment 1. Similarly, the effect of experimental treatments in exp. 1 and their interaction on the number of emerged adults and the number of surviving larvae at day 35 was tested. Likewise, the effects of temperature, eutrophication and their interaction in exp. 2 were tested on larval development rate and the percentage of larvae that survived until emergence. Temperature was a categorical variable with three levels in exp. 1 (22.7, 25.3 and 28.1°C) and four levels in exp. 2 (22.1, 24.1, 26.1 and 26.8°C). Predator presence (exp. 1) and eutrophication (exp. 2) were binomial variables representing top-down and bottom-up effects. As shown in S2 Fig, each treatment in both experiments was included only once in each row and column. Row and column could therefore be included in the model as random effects [35]. To detect the most important abiotic predictors for the abundance of *Cx pipiens* in exp. 2, a generalized linear regression model was used. The full model consisted of the following non-collinear main effects: temperature, EC and chlorophyll A. Significant effects (P < 0.05) were entered in the models in a forward stepwise fashion, starting with the most significant term. To meet assumptions of normality and homogeneity of variances, all response variables were square root transformed prior to analysis. Statistics were carried out in Statistica 7.0 and graphs were made in Sigmaplot 13.0.

## Results

### Experiment 1: Effects of predators on larval population parameters

#### Emergence of adult *Cx*. *pipiens*

**Fig 1:**
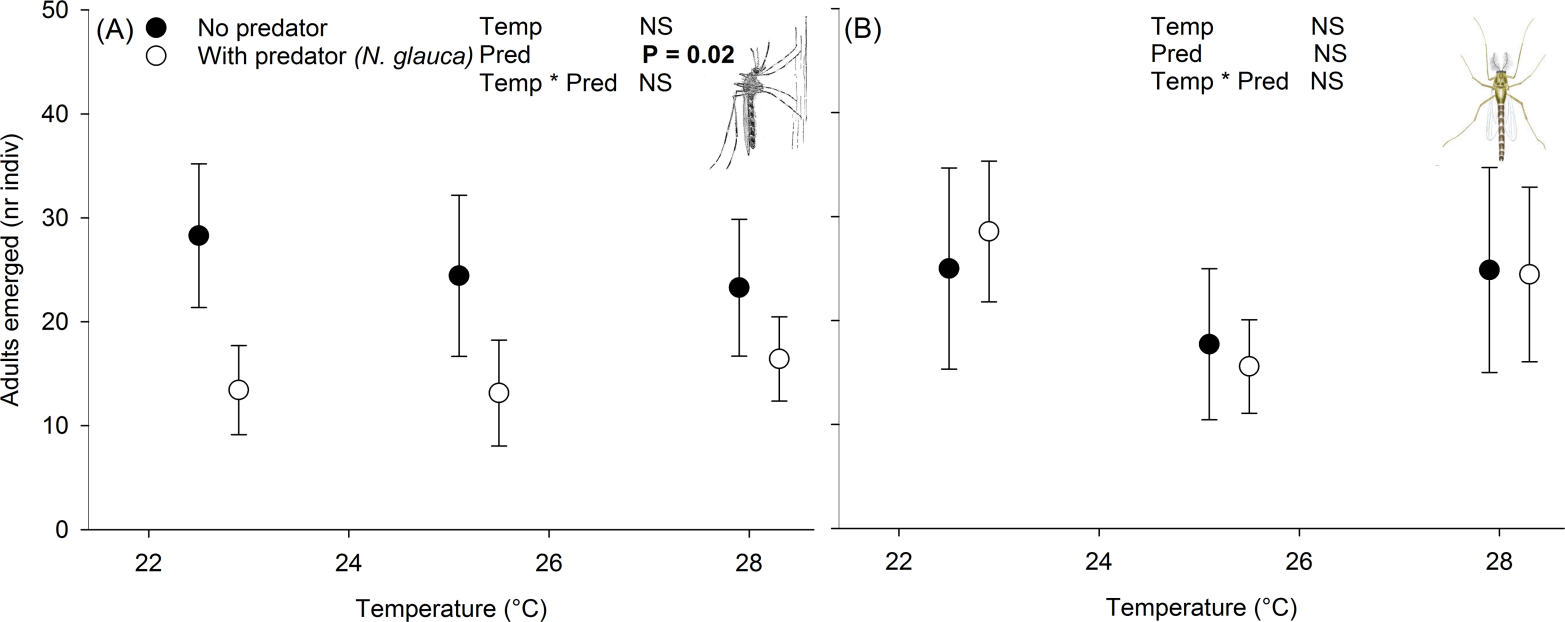
Emergence of adult *Cx*. *pipiens* (A) *and Ch*. *riparius* (B) from mesocosms with and without *N*. *glauca*, under different temperature regimes. Stats shown in upper right corner of each panel were carried out on square root-transformed numbers. NS: P > 0.05.

The presence of *N*. *glauca* decreased the number of emerging adult *Cx*. *pipiens* after 36 days (F_(1,36)_5.7, P = 0.02; Fig 1). There was no significant effect of temperature (F_(2,36)_0.9, P = 0.4) and no significant interaction between temperature and predator abundance (F_(2,36)_0.3, P = 0.7). Taken across temperature treatments, the presence of *N*. *glauca* was associated with an average reduction in the mean number of adults emerged by 11±2.3 individuals (29–52% decrease).

#### Effects on survival of *Cx*. *pipiens* larvae

The presence of *N*. *glauca* negatively affected the number of surviving mosquito larvae in the mesocosms after terminating the experiment after 36 days (F_(1,36)_7.0, P = 0.01; Table 1). The presence of *N*. *glauca* was associated with an average reduction of 34±13 surviving larvae (32–73% decrease). We found no significant effect of temperature on the number of surviving mosquito larvae (Table 1).

#### Effects on *Cx*. *pipiens* pupae

The presence of *N glauca* had a strong negative effect on the number of surviving *Cx*. *pipiens* pupae (F_(1,36)_6.9; P = 0.02). *N*. *glauca* seemed more effective in suppressing pupae numbers at low temperatures than at higher temperatures, but this effect was not significant (Table 1). Across temperature treatments, the presence of *N*. *glauca* was associated with an average reduction of 8 (±2) surviving pupae (27–89% decrease; Table 1).

#### Effects on *Ch*. *riparius* larvae and adults

In presence of *N*. *glauca*, we found a higher number of *Ch*. *riparius* larvae at the end of the experiment (137% increase) (F_(1,35)_8.9, P = 0.005), but we found no effect on the number of emerging adults (F_(1,35)_0.12, P = 0.7). There was a weak and significant positive relationship between the number of emerged *Ch*. *riparius* and *Cx*. *pipiens* (Pearson’s r^2^ = 0.11; P = 0.03), but this relationship was not found for larvae (Pearson’s r^2^ = 0.0, P = 0.6). Also, we found no significant effect of temperature.

### Experiment 2: Effects of eutrophication on larval development rate, mortality and emergence

#### Experimental conditions

We found strong effects of eutrophication on biotic and abiotic water parameters (EC, pH, chlorophyll A) but no effect of temperature (Table 2). In mesocosms with added nutrients, EC values were significantly lower than in mesocosms without nutrients (F_(1,40)_ 46.6; P<0.001; Table 2), and in mesocosms with added nutrients, pH values were higher (F_(1,40)_ 49.6; P<0.001; Table 2).

**Table 2:** Overview of the temperature treatments, abiotic variables and chlorophyll A concentrations in experiment 2 ± standard error (SE). EC (mV) = Electro conductivity in millivolt (mV). Different letters indicate significant differences between treatments at α = 0.05.

Nutrient treatment	Mean temp (°C)	SE		Temp 6 AM (°C)	SE		Temp 12 AM (°C)	SD		EC (mV)	SE		pH	SE		Chl A (mg/l)	SE	
With NPK	22.15	± 0.57	a	19.78	± 0.16	a	23.34	0.19	a	-79.17	± 6.45	a	8.52	± 0.12	a	0.089	± 0.01	a
	23.47	± 0.63	b	20.30	± 0.37	a	25.05	0.35	b	-64.80	± 11.39	a	8.25	± 0.20	a	0.084	± 0.01	a
	25.30	± 0.27	c	19.52	± 0.24	a	28.19	0.52	c	-66.28	± 14.62	a	8.26	± 0.24	a	0.123	± 0.02	a
	26.21	± 0.44	d	19.99	± 0.33	a	29.32	0.38	d	-56.97	± 12.17	a	8.26	± 0.12	a	0.053	± 0.00	b
No NPK	21.75	± 0.61	a	19.85	± 0.08	a	22.70	0.31	a	-128.10	± 12.37	b	9.39	± 0.24	b	0.046	± 0.00	b
	23.38	± 0.48	b	19.64	± 0.41	a	25.26	0.09	b	-126.22	± 9.43	b	9.40	± 0.16	b	0.030	± 0.00	b
	25.18	± 0.38	c	20.01	± 0.24	a	27.76	0.19	c	-113.73	± 10.78	b	9.13	± 0.20	b	0.041	± 0.01	b
	26.09	± 0.54	d	20.03	± 0.73	a	29.12	0.41	d	-107.35	± 6.25	b	9.02	± 0.12	b	0.047	± 0.00	b

Mesocosms with nutrients had twofold higher chlorophyll A concentrations (F_(1, 40)_11.051; P = 0.002; Table 2; S5 Fig), but there was no effect of temperature on chlorophyll A (F_(3,40)_ 0.9; P = 0.4; Table 2). This indicates a positive effect of nutrient additions on algal growth, which was confirmed by a noticeable decrease in water clarity (S5 Fig).

#### Effects of temperature and eutrophication on *Cx*. *pipiens* and *Ch*. *riparius*

**Fig 2:**
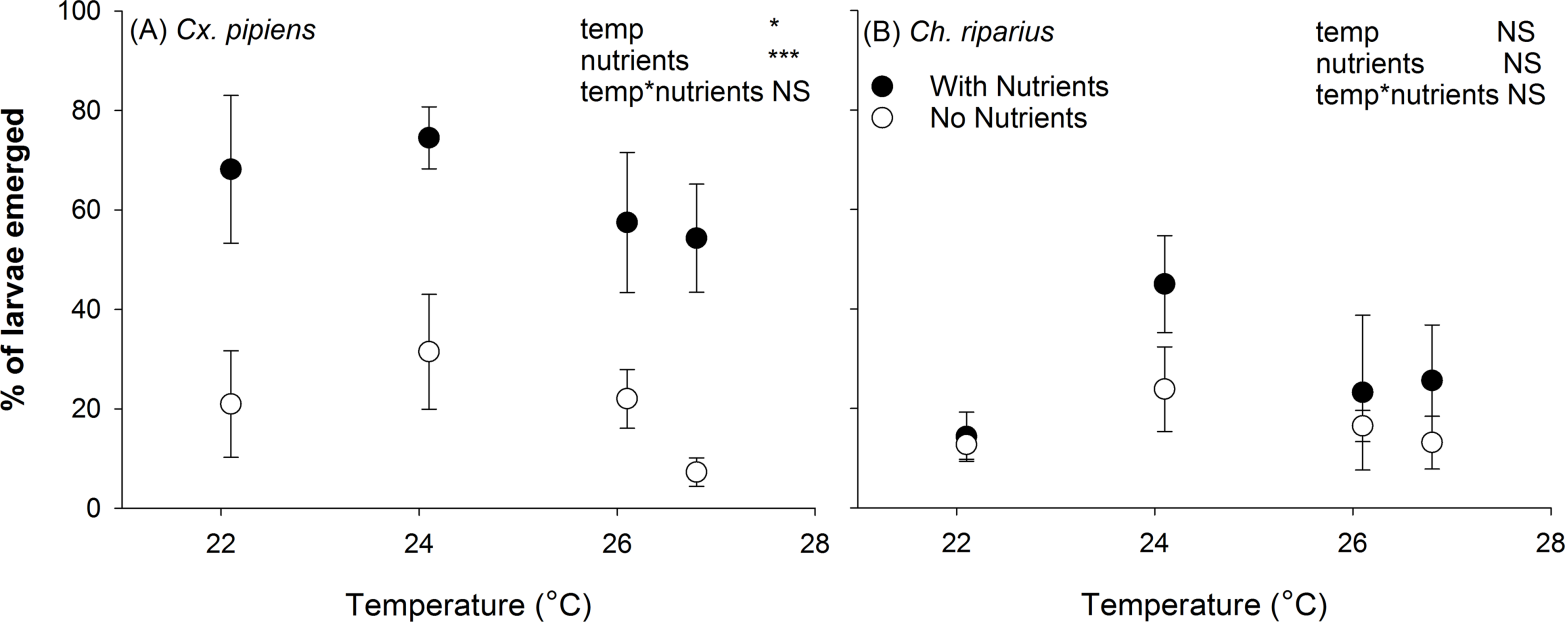
Effects of nutrient additions and temperature on the percentage of larvae that emerged as adults for (A) *Cx*. *pipiens* and (B) *Ch*. *riparius*. Model results are included in upper right corner of each panel. Stars indicate significance level: *** P < 0.001; *: 0.01 < P < 0.05; NS: P > 0.05.

Two mesocosms yielded no emerging male mosquitoes and four mesocosms had no emerging female mosquitoes, all of which belonged to the treatment with no added nutrients and the highest temperature treatment (30°C). We found a small but significant effect of temperature on survival of *Cx*. *pipiens* (F_(3,38)_3.18; P = 0.035), where the percentage of emerged adult *Cx*. *pipiens* was highest at 24.1°C (62%) and lowest at 26.8°C (33.4%; Fig 2). For *Cx*. *pipiens*, nutrient additions increased the fraction of larvae that survived until emergence by 81% (F_(1,38)_30.4; P<0.001; Fig 2). This positive effect was not found for *Ch*. *riparius* (F(_1,40)_0.6; P = 0.4).

**Fig 3:**
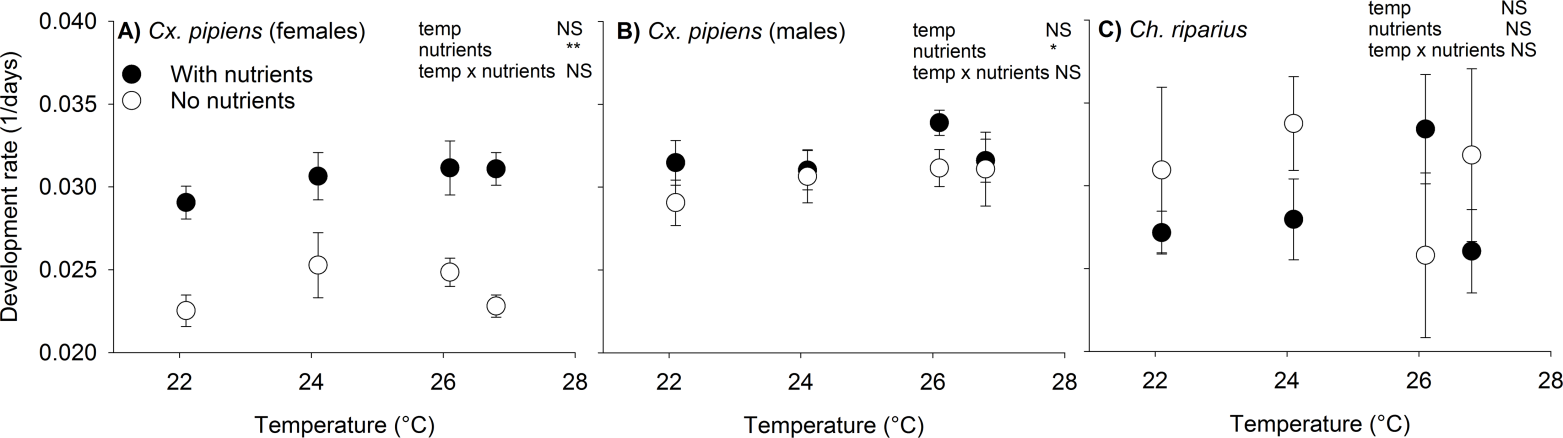
Effect of nutrients and temperature on development rate (1/(#days between egg and adult emergence)) of female (A) and male (B) *Cx*. *pipiens* mosquitoes and C) *Ch*. *riparius* adults. Model results are included in upper right corner of each panel. Stars indicate significance level: *** P < 0.001; **: 0.001< P <0.01; *: 0.01 < P < 0.05; NS: P > 0.05.

Temperature had no significant effect on the development rate of *Cx*. *pipiens*, but nutrients had a small effect. Females emerged more than two days earlier in the presence of nutrients (38.2 days vs 40.7 days; F_(1,34)_8.41, P = 0.006; Fig 3A). Although males developed faster than females, neither nutrient additions (with nutrients: 31.6 days vs without nutrients: 33.7 days; F_(1,38)_2.16, P = NS) nor temperature had a significant effect on relative development rate in males vs. females (Fig 3B). We also found no effect of temperature or nutrient treatment on the development rate of *Ch*. *riparius* (nutrients: F_(1,40)_0.7; P = 0.4; temperature: F_(3,40)_2.4; P = 0.07; Fig 3C).

EC was the strongest abiotic predictor of the number of emerging adult mosquitoes (Forward Stepwise Regression Model; F_(1,40)_9.0, P = 0.005), and temperature was the second strongest predictor (F_(1,40)_6.2, P = 0.02). We found a linear relationship between EC and adult emergence of *Cx*. *pipiens*: more adults emerged at lower EC, indicating the positive effect of eutrophication on survival (R^2^ = 0.18, P = 0.002; S5 Fig). No such relationship was found between survival and Chl A. A similar analysis on the number of emerged *Ch*. *riparius* showed that none of the measured abiotic variables significantly explained the adult emergence of *Ch*. *riparius* (S5 Fig).

## Discussion

While numerous studies have examined the effect of larval rearing temperature on adult mosquito fitness, far fewer studies have examined how temperature in conjunction with bottom-up and top-down factors affect larval survival and development rates. Previous work on the ecological drivers of mosquitos were carried out under highly controlled lab conditions and with a limited number of temperature regimes [21,22,36]. Our findings in more ecologically realistic settings suggest that these ecological drivers act in addition to temperature to cause significant impacts on mosquito survival and development rates.

The presence of predators negatively impacts various stages of mosquito development [18,31,37,38]. Our results suggest these results also occur in natural settings (Fig 1) and preliminary experiments showed that *N*. *glauca* increases mortality in mosquito larval by 10–20 fold per day (S1 Table, S1 Text, S3 Fig), which was higher than for *Orthetrum cancellatum*, a common dragonfly species in ponds. The high feeding efficiency of *N*. *glauca* could be due to the cursorial hunting behaviour of this species, compared to for example larvae of dragonflies that tend to forage on top of the sediment [29]. The presence of *N*. *glauca* disproportionally affected mosquito larvae (*Cx*. *pipiens)* over controphic species (*Ch*. *riparius*), suggesting a possible feeding preference for mosquitoes, which is in line with previous observations [18]. Other studies on feeding preference of dragonfly larvae *(Pantala hymenaea)* showed a slight preference for Chironomidae over Culicidae [39], indicating a possible difference between Odonata and Hemiptera species. These results thus indicate apparent competition between Chironomidae and Culicidae, which is important for potential use of predators in mosquito and mosquito-borne disease control [17,37], because prey specificity is an important component of biological control. Additional information is required on the probability that invertebrate predators such as *N*. *glauca* effectively colonize existing ponds [15] and how predator density affect colonization rates.

Breeding habitats of mosquitoes, most notably temporary ponds, often have high levels of eutrophication [40,41]. In our experiment, nutrient additions were associated with both higher survival and development rates for *Cx*. *pipiens*, following results in laboratory studies [14,28]. These increases were likely caused by the increase in food availability in the water column. Mosquitoes consume microorganisms and our measurements of EC and chlorophyll A levels suggest an increase in microorganisms in the mesocosms with added nutrients. Our results also show that these positive effects were only observed for *Cx*. *pipiens* and not for *Ch*. *riparius*. This may be related to the fact that *Cx*. *pipiens* ingests food items (bacteria, detritus) from the water column where positive effects of nutrient additions operate directly, whereas *Ch*. *riparius* larvae feeds in or on top the sediment, where effects of nutrient additions may have a much smaller effect.

The observed strong effects of predation and nutrient addition were larger than the effect of temperature on both survival and development rates. Whereas numerous studies have illustrated the importance of temperature on larval development and other life history parameters [e.g., 8–14], we observed only a minor effects of temperature. Two factors likely contribute to the observed marginal effects of temperature. First, the range of temperatures considered in this study (22.7–28.1°C) is smaller than the range possible in highly controlled, laboratory settings. In laboratory studies that consider similar temperature ranges, temperature was also observed to have a marginal effect [8,10]. The largest effects of temperature generally occur at extreme values, with most studies using temperature extremes of < 15°C and > 30°C [8,10]. Also in our experiment 2, the largest effects were found at the highest temperature regime in absence of eutrophication. The temperature range used in our experiment is based on current estimates (2–4°C) of climate change scenarios [42] and reflects a realistic range of temperature values in Europe. Second, in using outdoor experiments and fluctuating temperature regimes, the conditions associated with these temperature treatments are different from laboratory controlled settings. For example, other biotic and abiotic factors co-vary with temperature (e.g. cyanobacterial growth [43], fungal pathogens [44]) and the consequence of constant compared to fluctuating temperatures remains unknown. Therefore, although the exact mechanisms driving the lack of response to temperature is unclear, the data presented here shows that the effect of temperature in this range was marginal compared to other ecological drivers of mosquito populations.

### Conclusion and implications

In conclusion, our results suggest that, in addition to temperature, ecological bottom-up (nutrient availability) and top-down (predation pressure) drivers can have strong impacts on mosquito life history parameters. As such, this study presents a case to consider local anthropogenic stressors in concert with climatological conditions to obtain an improved understanding of the factors driving mosquito populations. Our study may have implications for understanding mosquito-borne disease risk. By showing that mosquito survival and development rates are strongly driven by anthropogenic pressures related to global change, our results highlight two potentially important mechanisms driving spatial variation in vector abundance: eutrophication and biodiversity loss. Variation in mosquito abundance is one potentially important driver of variation in disease transmission [12,45], with consequences for the size and speed of an outbreak [46]. Knowledge of the mechanisms driving variation in mosquito abundance in natural settings will be important for managing the disease risks associated with future environmental change.

## Supporting information

S1 Table Mortality rates of 4^th^ instar *Cx*. *pipiens* in absence and presence of the two predators (*N*. *glauca* and *O*. *cancellatum*). Different letters indicate significant differences at α = 0.05. For description of methods, see S1 Text.
(DOCX)

S1 Fig Average temperature (±SD) between 18 August and 15 October 2016 at the KNMI station of Voorschoten, approx. 3.5 km from the experimental garden at the same altitude. The Y-axis indicates the hour that temperatures were logged on a 24-hr scale. Arrows indicate the times when temperature measurements in the mesocosms were taken; grey area indicates the time period that the heaters were switched on.
(DOCX)

S2 Fig Experimental design of experiment 1 (top panel) and 2 (bottom panel). The predation treatment in the first experiment was visualized using a symbol of a small individual *Notonecta glauca*; eutrophication in the second experiment was indicated through a star.
(DOCX)

S3 Fig Effect of (a) absence of predators, (b) *N*. *glauca* and (c) *O*.*cancellatum* on larval survival at different initial larval densities. For description of methods, see S1 Text.
(DOCX)

S4 Fig Differences in algal load at the termination of the second mesocosm experiment. Top left picture shows a mesocosm from the nutrient addition treatment, the picture on the Top right picture shows a mesocosm with no added nutrients. The lower panel shows a relative measure of the chlorophyll A concentration in the different treatments and temperatures; * P<0.05; + P<0.1; NS not significant.
(DOCX)

S5 Fig Relationship between abiotic parameters and adult emergence of *Cx*. *pipiens* and *Ch*. *riparius*. Only for the former species, we found a significant Pearson’s r between adult emergence and abiotic parameters (see legend).
(DOCX)

S1 Text Methods concerning a small lab experiment for quantification of predation efficiencies of *N*. *glauca* and *Orthetrum cancellatum* (results shown in S1 Fig and S1 Table). (DOCX)

S2 Text Supplemental file containing the data of experiment 1, 2 as well as the supplemental figures and tables. (XLSX)
