# Supplementary material for: Eutrophication and predator presence overrule the effects of temperature on mosquito survival and development
Source: PLoS Negl Trop Dis. 2018 Mar 26;12(3):e0006354. doi: 10.1371/journal.pntd.0006354 (PMC5898759; doi:10.1371/journal.pntd.0006354)
Supplement: S1 Table — Different letters indicate significant differences at α = 0.05. For description of methods, see S1 Text. (DOCX) [file pntd.0006354.s001.docx]

S1 Table. Mortality rates of 4^th^ instar *Cx. pipiens* in absence and presence of the two predators (*N. glauca* and *O. cancellatum*). Different letters indicate significant differences at ɑ=0.05.

| **Treatment** | 5 indiv l^-1^ | Increase (rel. to cont) | 10 indiv l^-1^ | Increase (rel. to cont) | 20 indiv l^-1^ | Increase (rel. to cont) |
| --- | --- | --- | --- | --- | --- | --- |
| Control | -0.033a |  | -0.042 |  | -0.083 |  |
| *O. cancellatum* | -0.433b | *13.1* | -0.317 | *7.5* | -0.301 | *3.6* |
| *N. glauca* | -0.483b | *14.7* | -0.408 | *9.7* | -0.354 | *4.3* |
