# Supplementary material for: Eutrophication and predator presence overrule the effects of temperature on mosquito survival and development
Source: PLoS Negl Trop Dis. 2018 Mar 26;12(3):e0006354. doi: 10.1371/journal.pntd.0006354 (PMC5898759; doi:10.1371/journal.pntd.0006354)
Supplement: S1 Fig — 3.5 km from the experimental garden at the same altitude. The Y-axis indicates the hour that temperatures were logged on a 24-hr scale. Arrows indicate the times when temperature measurements in the mesocosms were taken; grey area indicates the time period that the heaters were switched on. (DOCX) [file pntd.0006354.s002.docx]

S1 Figure. Average temperature (±SD) between 18 August and 15 October 2016 at the KNMI station of Voorschoten, approx. 3.5 km from the experimental garden at the same altitude. The Y-axis indicates the hour that temperatures were logged on a 24-hr scale. Arrows indicate the times when temperature measurements in the mesocosms were taken; grey area indicates the time period that the heaters were switched on.
