# Supplementary material for: Eutrophication and predator presence overrule the effects of temperature on mosquito survival and development
Source: PLoS Negl Trop Dis. 2018 Mar 26;12(3):e0006354. doi: 10.1371/journal.pntd.0006354 (PMC5898759; doi:10.1371/journal.pntd.0006354)
Supplement: S2 Fig — The predation treatment in the first experiment was visualized using a symbol of a small individual Notonecta glauca; eutrophication in the second experiment was indicated through a star. (DOCX) [file pntd.0006354.s003.docx]

*

*

*

*

*

*

*

*

*

*

*

*

*

*

*

*

*

*

*

*

*

*

*

*

*

22.7 ^0^C

25.3 ^0^C

28.1 ^0^C

Predator treatment

1 indiv *Notonecta glauca*

24.1 ^0^C

26.1 ^0^C

28.8 ^0^C

Eutrophication treatment

22.1 ^0^C

Experiment 1

Experiment 2

S2 Figure. Experimental design of experiment 1 (top panel) and 2 (bottom panel). The predation treatment in the first experiment was visualized using a symbol of a small individual *Notonecta glauca*; eutrophication in the second experiment was indicated through a star.
