# Supplementary material for: Eutrophication and predator presence overrule the effects of temperature on mosquito survival and development
Source: PLoS Negl Trop Dis. 2018 Mar 26;12(3):e0006354. doi: 10.1371/journal.pntd.0006354 (PMC5898759; doi:10.1371/journal.pntd.0006354)
Supplement: S3 Fig — Effect of (a) absence of predators, (b) N. glauca and (c) O.cancellatum on larval survival at different initial larval densities. For description of methods, see S1 Text. (DOCX) [file pntd.0006354.s004.docx]

S4 Figure. Effect of predators on survival of Cx. pipiens larvae: (a) absence of predators, (b) presence of *N. glauca* and (c) presence of *O.cancellatum* at different initial larval densities.
