# Supplementary material for: Eutrophication and predator presence overrule the effects of temperature on mosquito survival and development
Source: PLoS Negl Trop Dis. 2018 Mar 26;12(3):e0006354. doi: 10.1371/journal.pntd.0006354 (PMC5898759; doi:10.1371/journal.pntd.0006354)
Supplement: S4 Fig — Top left picture shows a mesocosm from the nutrient addition treatment, the picture on the Top right picture shows a mesocosm with no added nutrients. The lower panel shows a relative measure of the chlorophyll A concentration in the different treatments and temperatures; * P<0.05; + P<0.1; NS not significant. (DOCX) [file pntd.0006354.s005.docx]

(b)

(a)


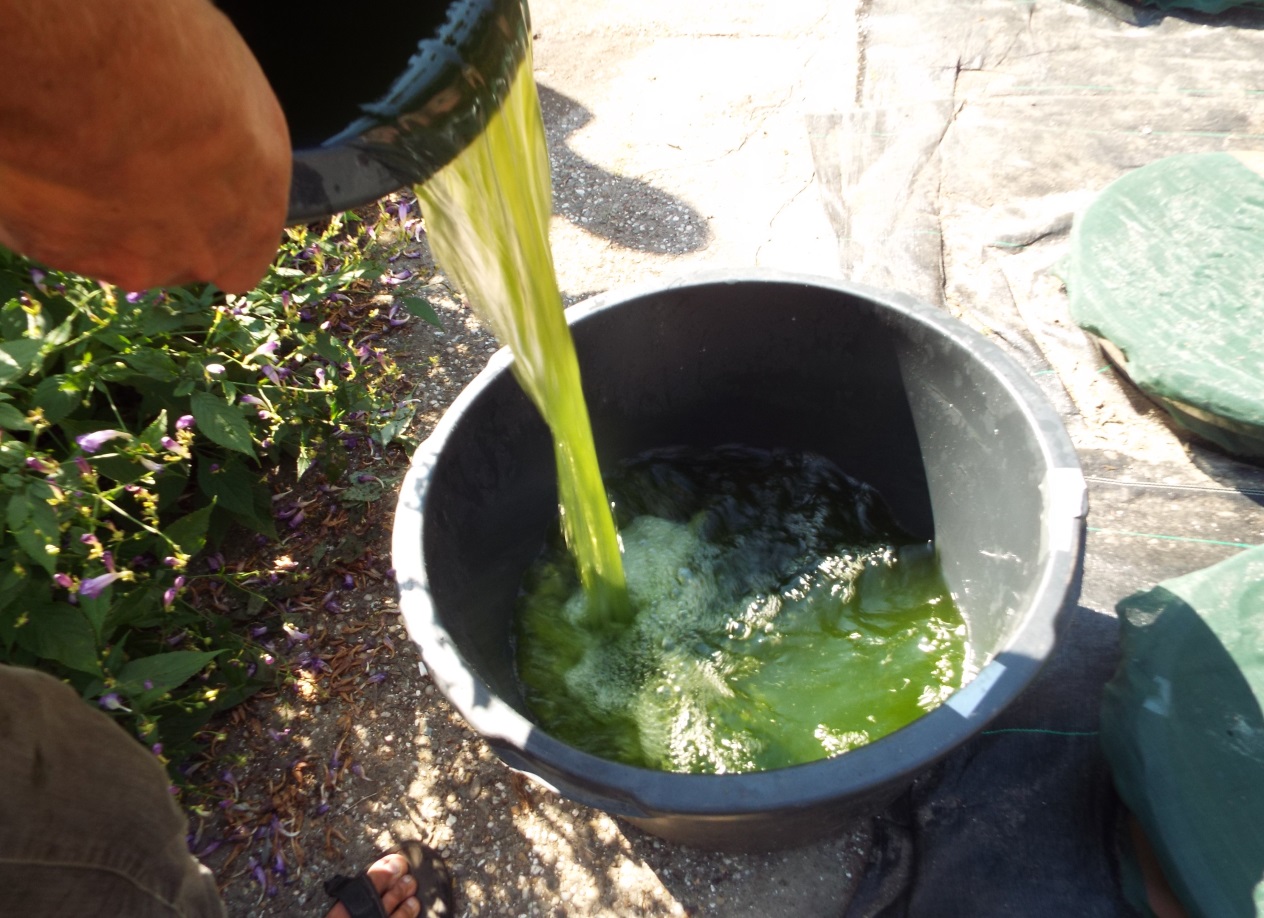

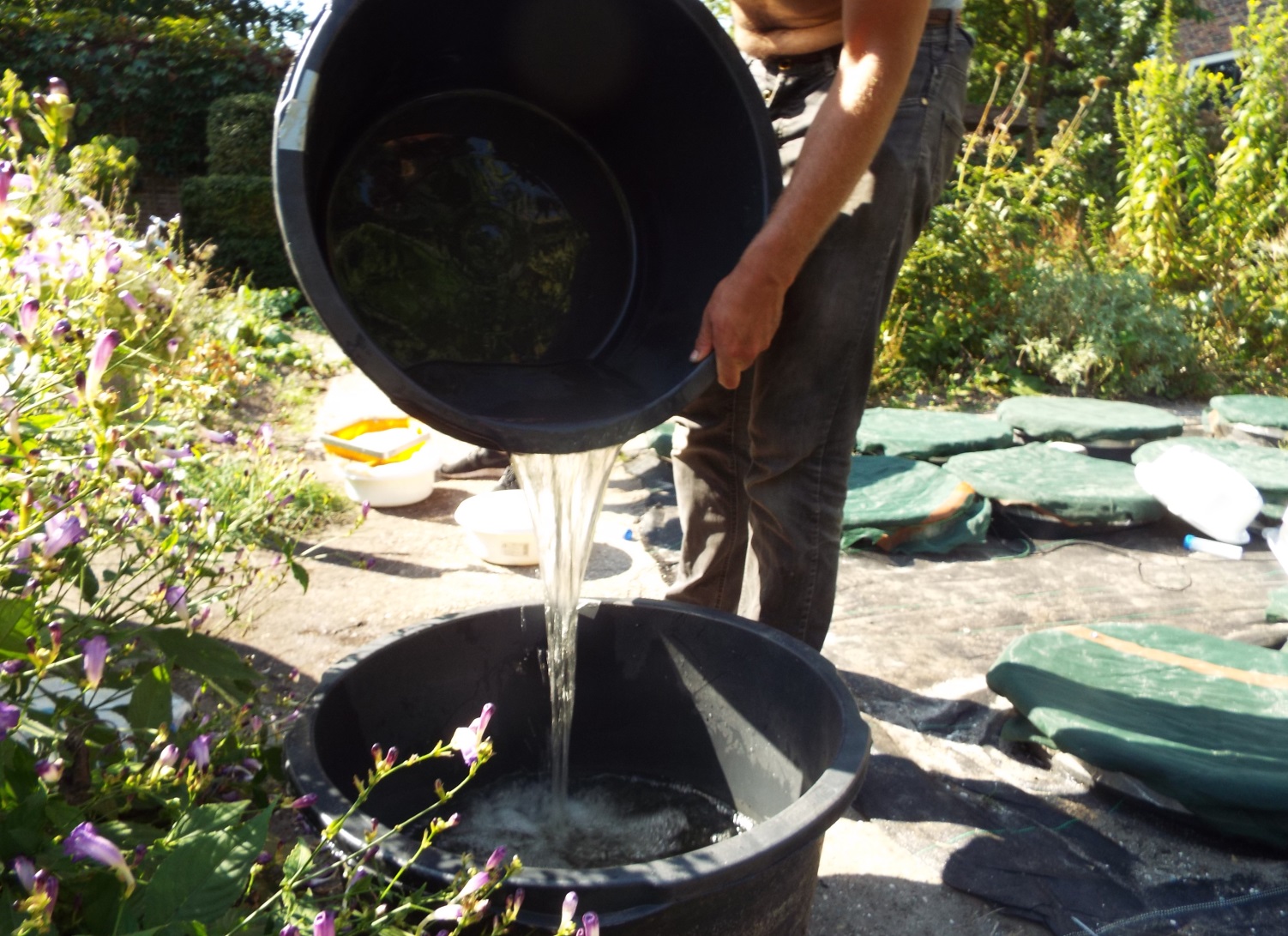


(c)

S4 Figure. Differences in algal load at the termination of the second mesocosm experiment. A mesocosm from the nutrient addition treatment is shown on the top left (a); the picture on the top right picture shows a mesocosm with no added nutrients (b). The lower panel (c) shows a relative measure of the chlorophyll A concentration in the different treatments and temperatures; * P<0.05; + P<0.1; NS not significant.
