# Supplementary material for: Eutrophication and predator presence overrule the effects of temperature on mosquito survival and development
Source: PLoS Negl Trop Dis. 2018 Mar 26;12(3):e0006354. doi: 10.1371/journal.pntd.0006354 (PMC5898759; doi:10.1371/journal.pntd.0006354)
Supplement: S5 Fig — Only for the former species, we found a significant Pearson’s r between adult emergence and abiotic parameters (see legend). (DOCX) [file pntd.0006354.s006.docx]

S5 Figure. Relationship between abiotic parameters and adult emergence of *Cx. pipiens* and *Ch. riparius.* Only for the former species, we found a significant Pearson’s r between adult emergence and abiotic parameters (see legend).
