# Supplementary material for: Eutrophication and predator presence overrule the effects of temperature on mosquito survival and development
Source: PLoS Negl Trop Dis. 2018 Mar 26;12(3):e0006354. doi: 10.1371/journal.pntd.0006354 (PMC5898759; doi:10.1371/journal.pntd.0006354)
Supplement: S1 Text — (DOCX) [file pntd.0006354.s007.docx]

S1 Text

Methods concerning a small lab experiment for quantification of predation efficiencies of *N. glauca* and *Orthetrum cancellatum* (results shown in Figure S1 and Table S1).

A small side experiment was set up to determine the foraging rate of different mosquito predators: the dragonfly larvae *Orthetrum cancellatum* and the backswimmer *N. glauca*. Different numbers of fourth instar larvae (10, 20, 40) of *Cx. pipiens* were placed in 5 L transparent containers (hereafter: microcosms) containing 2 L of dechlorinated tap water. On day 1, 4 hours after placing the larvae in the microcosms, larvae were either exposed to a dragonfly larvae (n=3), a backswimmer (n=3) or no predator (n=3). The number of remaining larvae was counted daily in each of the microcosms for 3 days.

Results

Predator presence had an important effect on survival of larval *Cx. pipiens.* Predation by *N*. *glauca* and *O. cancellatum* resulted in an almost 100% decrease in larval abundance at the lowest density (5 larvae/litre), 3 days after the experiment started. In general, mortality rates were 13-14 times higher in containers with predation at the lowest density of larvae, but were only 3-4 times higher at the highest larval density (Figure S3 and Table S1 below).
